# Short-term changes of right ventricular-pulmonary artery coupling after TAVR and their association with clinical outcomes

**DOI:** 10.3389/fcvm.2025.1649880

**Published:** 2025-12-04

**Authors:** Xiaoke Ma, Chi Zhou, Yuefeng Ju, Zongyi Xia, Zhexun Lian

**Affiliations:** Department of Cardiology, The Affiliated Hospital of Qingdao University, Qingdao, Shandong, China

**Keywords:** TAVR, RV-PA coupling, survival analysis, echocardiography, right ventricular function

## Abstract

**Background:**

The prognostic significance of the trajectories of right ventricular-pulmonary artery (RV-PA) coupling after transcatheter aortic valve replacement (TAVR) remains underexplored.

**Objectives:**

This study aimed to (1) characterize the short-term changes of RV-PA coupling (assessed by TAPSE/PASP ratio) from baseline to approximately 3 months after TAVR, and (2) analyze its association with clinical outcomes, including all-cause mortality.

**Methods:**

In this retrospective analysis, a total of 210 severe AS patients undergoing TAVR from July 2017 to April 2023 were included based on complete information and were divided into four groups according to the longitudinal changes in RV-PA coupling status. RV-PA coupling was defined as the TAPSE/PASP ratio (normal coupling: >0.55; uncoupling: ≤0.55). Observations included all-cause mortality, heart failure rehospitalization, and NYHA functional class.

**Results:**

Among those with normal RV-PA coupling at baseline (*n* = 129), 87.6% (113/129) maintained preserved coupling at follow-up (Group 1), while 12.4% (16/129) developed new-onset uncoupling (Group 3). Of those with baseline RV-PA uncoupling (*n* = 81), 72.8% (59/81) achieved normalization (Group 2), whereas 27.2% (21/81) exhibited persistent uncoupling (Group 4). Group 4 was associated with higher all-cause mortality (45.5% vs. 4.4%, *P* < 0.05). Multivariable Cox regression analysis identified persistent RV-PA uncoupling as an independent predictor of mortality (HR: 5; 95% CI: 1.29–19.35; *P* = 0.02).

**Conclusion:**

Persistent RV-PA uncoupling after TAVR independently predicts adverse outcomes. Serial assessment of RV-PA coupling enhances risk stratification, supporting tailored post-procedural management.

## 1 Introduction

Aortic stenosis (AS) represents a growing health challenge in aging societies, with prevalence escalating in parallel with demographic shifts toward the older population (1, 2). Since the initial human application of transcatheter aortic valve replacement (TAVR) in 2002 (3), it has evolved into a cornerstone therapy for severe AS. Several randomized controlled trials establish TAVR as non-inferior or even superior to surgical aortic valve replacement (SAVR) for short-term mortality and major adverse events in low-to-intermediate risk cohorts (4–6). Right ventricular-pulmonary artery (RV-PA) coupling quantifies right ventricular energy transfer efficiency to the pulmonary vasculature, with the tricuspid annular systolic displacement to pulmonary artery systolic pressure ratio (TAPSE/PASP) serving as its echocardiographic surrogate. Both right ventricular dysfunction (7) and pulmonary hypertension (8) have been identified as negatively impacting the prognosis of patients undergoing TAVR. However, only a few studies have comprehensively assessed the synergistic effect of RV-PA coupling on the prognosis of TAVR. Previous research has confirmed that baseline RV-PA uncoupling is associated with postoperative all-cause mortality in patients treated with TAVR (9, 10). A multicenter retrospective cohort study in 2023 demonstrated that post-TAVR RV-PA coupling normalization associates with a favorable prognosis comparable to baseline-preserved coupling, whereas uncoupling progression elevates mortality (11). While earlier studies concentrated on baseline RV-PA coupling, the dynamic changes post-TAVR and its relationship with non-mortality outcomes (e.g., quality of life, functional capacity, and readmission rate) remain underexplored. We aimed to evaluate the changes in RV-PA coupling before and after TAVR in patients with severe AS and to analyze its association with clinical outcomes, including all-cause mortality.

## 2 Methods

We retrospectively analyzed hospitalized patients diagnosed with severe aortic stenosis (peak aortic velocity >4 m/s, mean transaortic pressure gradient >40 mmHg, or aortic valve area <1.0 cm^2^) who underwent TAVR from July 2017 to April 2023 at the Affiliated Hospital of Qingdao University. The exclusion criteria were as follows: (1) incomplete echocardiographic data; (2) lost to follow-up; (3) undergoing a combined cardiac procedure; and (4) bicuspid aortic valve morphology. After applying the exclusion criteria, 210 patients were included in the final analysis. Patients' clinical information and test and examination data are collected through the inpatient electronic medical record system, and follow-up visits are conducted through patients' reserved telephone numbers and outpatient clinics. The study was conducted in accordance with the principles of the Helsinki Declaration. This article was approved by the Ethics Committee of the Affiliated Hospital of Qingdao University (ethics number: QYFY WZLL 30202).

All echocardiographic examinations and measurements were performed on a Philips EPIQ 7C system using its integrated vendor software. The retrospective nature of our study precluded a formal assessment of inter-observer variability for the echocardiographic measurements. However, all measurements were conducted by highly trained personnel following standardized protocols to minimize variability.

Routine transthoracic echocardiography was performed at two time points: (1) Baseline: approximately 7 days prior to the TAVR procedure; (2) Post-procedure: at scheduled follow-up visit approximately 3 months (median 92.5 days) after TAVR. Previous studies have shown that TAPSE/PASP >0.55 can be considered as normal RV-PA coupling (12), so we defined TAPSE/PASP >0.55 as normal coupling and TAPSE/PASP ≤0.55 as uncoupling. Patients were categorized into four trajectory groups based on serial RV-PA coupling assessments: group 1, unchanged normal RV-PA coupling; group 2, recovered RV-PA coupling; group 3, new-onset RV-PA uncoupling; and group 4, persistent RV-PA uncoupling.

The Valve Center team comprehensively evaluated all patients before surgery, and the appropriate surgical access and valve type were selected based on the patient's cardiac function, aortic valve-related content, vascular anatomy, and puncture point. The alternative access routes included transapical, transaortic, or transsubclavian approaches, which were selected in cases with unsuitable iliofemoral anatomy for transfemoral access. The procedure was performed under general anesthesia and endotracheal intubation. A temporary pacemaker was placed into the right ventricle. Prosthetic valve implantation was performed under digital subtraction angiography (DSA). An intraoperative transesophageal ultrasound probe was placed to assess the condition of the prosthetic valve.

The primary outcome of this study was all-cause mortality. Secondary outcomes included heart failure rehospitalization and NYHA functional class. Mortality data and NYHA functional class were obtained through scheduled follow-up clinics and telephone interviews. Heart failure rehospitalization was defined as an unplanned hospital admission primarily for management of acute heart failure. All outcomes were assessed from the time of the index TAVR procedure (time zero). Follow-up at 1, 3, 6, and 12 months after TAVR and yearly thereafter. The database was locked for final analysis in May 2025.

Normally distributed continuous data are expressed as means with standard deviations (SD), while non-parametric variables are reported as medians with interquartile ranges (IQR). Group comparisons employed ANOVA, Kruskal–Wallis *H* test, or Mann–Whitney *U* test as appropriate, with *post-hoc* adjustments for multiple testing. Categorical variables were summarized as numbers and percentages and analyzed by Pearson's chi-square test or Fisher's exact test, followed by Bonferroni-adjusted pairwise comparisons. Kaplan–Meier analysis assessed survival outcomes, and the log-rank test compared groups. The association between RV-PA coupling groups and mortality was further quantified using univariable and multivariable Cox proportional hazards regression. *P* < 0.05 was considered significant for all tests. All statistical analyses were performed using SPSS 26 (IBM Corp., Armonk, NY) and R 4.4.3 (R Foundation for Statistical Computing, Vienna, Austria).

## 3 Results

**Figure 1:**
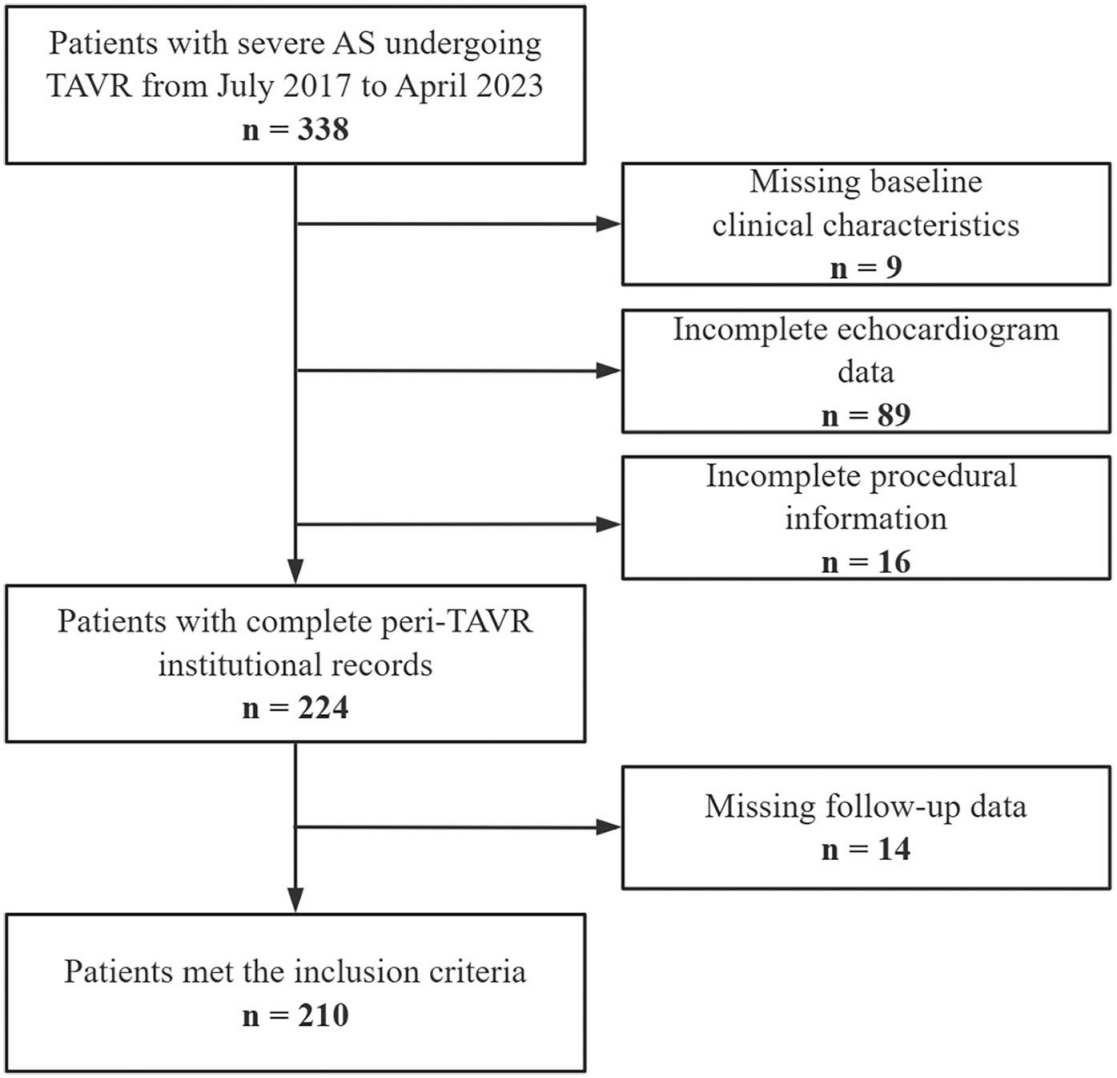
Study population and workflow.

Between July 2017 and April 2023, a total of 210 patients were enrolled in this study (Figure 1). Baseline characteristics are presented in Table 1. Group 4 exhibited a significantly higher prevalence of atrial fibrillation (27.3% vs. 8.8% in Group 1, *P* = 0.046), loop diuretic usage (27.3% vs. 8% in Group 1, *P* = 0.012), and longer index hospitalization duration (23 vs. 15 days in Group 1, *P* = 0.002).

**Table 1:** Baseline clinical characteristics of the study population stratified by RV-PA coupling status.

Characteristics	Group 1 unchanged normal RV-PA coupling (*n* = 113)	Group 2 recovered RV-PA coupling (*n* = 59)	Group 3 new-onset RV-PA uncoupling (*n* = 16)	Group 4 persistent RV-PA uncoupling (*n* = 22)	*X*^2^/*H*	*P* value
Age (years)	74 (70, 78)	74 (69, 77)	76.5 (70.75, 79.25)	75.5 (71.5, 77.75)	1.763	0.623
Female sex	42 (37.2%)	16 (27.1%)	6 (37.5%)	10 (45.5%)	2.931	0.402
BMI (kg/m^2^)	24.2 (22.8, 26.1)	23.5 (21.45, 26)	24.35 (22.57, 26.73)	20.7 (19.55, 24.85)	7.436	0.059
Diabetes	41 (36.3%)	18 (30.5%)	3 (18.8%)	5 (22.7%)	3.177	0.365
Hypertension	70 (61.9%)	30 (50.8%)	7 (43.8%)	10 (45.5%)	4.212	0.239
Hypercholesterolemia	29 (25.7%)	13 (22%)	4 (25%)	9 (40.9%)	2.820	0.420
Renal dysfunction	10 (8.8%)	8 (13.6%)	1 (6.2%)	4 (18.2%)	2.502	0.462
IHD	47 (41.6%)	24 (40.7%)	8 (50%)	10 (45.5%)	0.564	0.905
AF	10 (8.8%)	7 (11.9%)	4 (25%)	6 (27.3%)	7.599	**0** **.** **046**
Smoker	26 (23%)	22 (37.3%)	5 (31.2%)	6 (27.3%)	3.924	0.270
NYHA functional class III/IV	62 (54.9%)	37 (62.7%)	10 (62.5%)	15 (68.2%)	1.982	0.576
EuroSCORE II (%)	1.67 (1.05, 2.58)	1.89 (1.26, 2.94)	1.75 (1.44, 3.13)	2.49 (1.77, 3.62)	7.186	0.066
Hospitalization duration	15 (13, 20)	16 (14, 20)	17 (13.75, 22.25)	23 (16.25, 25)	14.86	**0** **.** **002**
Prehospital medication use						
ACEI/ARB/ARNI	41 (36.3%)	14 (23.7%)	6 (37.5%)	7 (31.8%)	2.998	0.392
Beta-blocker	30 (26.5%)	11 (18.6%)	3 (18.8%)	6 (27.3%)	1.752	0.625
MRA	5 (4.4%)	9 (15.3%)	2 (12.5%)	2 (9.1%)	6.522	0.065
Loop diuretic	9 (8%)	12 (20.3%)	4 (25%)	6 (27.3%)	10.283	**0** **.** **012**
Antiplatelet therapy	29 (25.7%)	17 (28.8%)	8 (50%)	4 (18.2%)	4.906	0.179
Oral anticoagulant	10 (8.8%)	5 (8.5%)	2 (12.5%)	4 (18.2%)	2.397	0.490

BMI, body mass index; IHD, ischemic heart disease; AF, atrial fibrillation; ACEI/ARB/ARNI, angiotensin-converting enzyme inhibitors/angiotensin receptor blockers/angiotensin receptor-neprilysin inhibitors; MRA, mineralocorticoid receptor antagonists; RV-PA, right ventricular-pulmonary artery.

Statistical significance at the 0.05 level is shown in bold type.

Echocardiographic evaluation was performed at approximately 3 months post-TAVR. 129 (61.4%) exhibited normal RV-PA coupling at baseline, of whom 113 (87.6%) maintained normal coupling postoperatively (Group 1), while 16 (12.4%) developed new-onset uncoupling (Group 3). Of the 81 patients with baseline RV-PA uncoupling, 59 (72.8%) achieved normalization (Group 2), whereas 22 (27.2%) remained uncoupled (Group 4). Preoperatively, patients with baseline RV-PA uncoupling (Groups 2 and 4) demonstrated lower LVEF, reduced TAPSE, and elevated PASP. Postoperatively, Patients with post-TAVR uncoupling (Groups 3 and 4) exhibited persistently higher PASP and higher rates of moderate-to-severe mitral regurgitation and tricuspid regurgitation in the postoperative period, whereas the valve regurgitation rate in group 2 decreased significantly postoperatively (Table 2).

**Table 2:** Baseline and postprocedural echocardiographic parameters of the study population stratified by RV-PA coupling status.

Echocardiographic parameters	Group 1 unchanged normal RV-PA coupling (*n* = 113)	Group 2 recovered RV-PA coupling (*n* = 59)	Group 3 new-onset RV-PA uncoupling (*n* = 16)	Group 4 persistent RV-PA uncoupling (*n* = 22)	*X*^2^/*H*	*P* value
Pre-TAVR						
LVIDd (mm)	4.7 (4.4, 5)	5.3 (4.75, 5.6)	4.65 (4.4, 4.92)	5.15 (4.5, 5.58)	24.595	**<0** **.** **001**
LVIDs (mm)	3.2 (2.9, 3.4)	3.8 (3.1, 4.3)	3 (2.9, 3.7)	3.45 (2.92, 4)	19.041	**<0** **.** **001**
LVPWd (mm)	1.1 (1, 1.2)	1.1 (1, 1.2)	1.1 (1, 1.2)	1.2 (1.12, 1.2)	3.952	0.267
IVSd (mm)	1.3 (1.2, 1.4)	1.3 (1.2, 1.4)	1.3 (1.27, 1.43)	1.3 (1.3, 1.4)	2.099	0.552
LVEF (%)	60 (57, 61)	48 (40.5, 59)	58 (56.5, 60)	53.5 (42.5, 60)	37.986	**<0** **.** **001**
Peak aortic velocity (m/s)	4.5 (4.2, 4.9)	4.6 (4.1, 5)	4.45 (4.1, 4.85)	4.75 (4.2, 5.1)	1.102	0.777
MTPG (mmHg)	49 (42, 62)	51 (41.5, 61)	52.5 (40, 58.5)	57.5 (45.25, 60.75)	0.597	0.897
AVA-D (cm^2^)	0.7 (0.6, 0.9)	0.65 (0.5, 0.8)	0.8 (0.7, 0.83)	0.8 (0.6, 0.8)	7.091	0.069
TAPSE (mm)	20 (20, 21)	19 (17, 20.5)	20 (20, 23)	19.5 (18, 20)	36.617	**<0** **.** **001**
PASP (mmHg)	28 (26, 31)	45 (40, 54.5)	31 (30, 33.25)	45 (40, 55.75)	135.691	**<0** **.** **001**
Moderate or severe AR	37 (32.7%)	25 (42.4%)	4 (25%)	12 (54.5%)	5.492	0.139
Moderate or severe MR	12 (10.6%)	27 (45.8%)	3 (18.8%)	11 (50%)	33.477	**<0** **.** **001**
Moderate or severe TR	4 (3.5%)	20 (33.9%)	0	9 (40.9%)	39.997	**<0** **.** **001**
Post-TAVR						
LVIDd (mm)	4.5 (4.3, 4.8)	4.8 (4.45, 5.1)	4.75 (4.38, 5.12)	4.6 (4.3, 5.05)	7.750	0.051
LVIDs (mm)	3 (2.7, 3.3)	3.2 (2.85, 3.7)	3.05 (2.8, 3.6)	3 (2.73, 3.5)	8.490	**0** **.** **037**
LVPWd (mm)	1.1 (1, 1.2)	1 (1, 1.2)	1.15 (1.07, 1.23)	1.2 (1.1, 1.3)	8.172	**0** **.** **043**
IVSd (mm)	1.2 (1.2, 1.3)	1.3 (1.2, 1.4)	1.2 (1.07, 1.4)	1.3 (1.2, 1.3)	0.439	0.932
LVEF (%)	60 (59, 61)	59 (55, 60)	60 (58.75, 61.25)	59.5 (56, 60)	18.068	**<0** **.** **001**
Peak aortic velocity (m/s)	2.2 (2, 2.5)	2.2 (1.9, 2.5)	2.3 (2.07, 2.88)	2.65 (2.12, 2.9)	8.037	**0** **.** **045**
MTPG (mmHg)	12 (8, 15)	10 (8, 16.5)	12.5 (9, 17)	14.5 (11.25, 19.5)	7.056	0.07
TAPSE (mm)	20 (20, 21)	20 (20, 21)	20 (19.5, 20.25)	20 (20, 20.75)	7.099	0.069
PASP (mmHg)	27 (25, 30)	29 (26, 31.5)	45 (41.25, 48.25)	43 (40, 48)	93.665	**<0** **.** **001**
Moderate or severe AR	1 (0.9%)	1 (1.7%)	1 (6.2%)	2 (9.1%)	6.211	0.063
Moderate or severe MR	3 (2.7%)	2 (3.4%)	6 (37.5%)	6 (27.3%)	26.387	**<0** **.** **001**
Moderate or severe TR	4 (3.5%)	6 (10.2%)	4 (25%)	6 (27.3%)	15.808	**<0** **.** **001**

LVIDd, left ventricular internal diameter in diastole; LVIDs, left ventricular internal diameter in systole; LVPWd, left ventricular posterior wall diastole; IVSd, interventricular septal thickness; LVEF, left ventricular ejection fraction; MTPG, mean transaortic pressure gradient; AVA-D, aortic valve area Doppler; TAPSE, tricuspid annular plane systolic excursion; PASP, pulmonary artery systolic pressure; AR, aortic regurgitation; MR, mitral regurgitation; TR, tricuspid regurgitation; TAVR, transcatheter aortic valve implantation; RV-PA, right ventricular-pulmonary artery.

Statistical significance at the 0.05 level is shown in bold type.

No significant intergroup differences emerged in access route, type of valve, pre-/post-dilation rates, permanent pacemaker implantation, or paravalvular leakage rates (Table 3).

**Table 3:** Procedural characteristics of the study population stratified by RV-PA coupling status.

Procedural characteristics	Group 1 unchanged normal RV-PA coupling (*n* = 113)	Group 2 recovered RV-PA coupling (*n* = 59)	Group 3 new-onset RV-PA uncoupling (*n* = 16)	Group 4 persistent RV-PA uncoupling (*n* = 22)	*X* ^2^	*P* value
Access route					3.579	0.211
Transfemoral	112 (99.1%)	57 (96.6%)	15 (93.8%)	22 (100%)		
Alternative	1 (0.9%)	2 (3.4%)	1 (6.2%)	0		
Type of valve					0.399	0.242
Self-expanding	101 (89.4%)	50 (84.7%)	15 (93.8%)	22 (100%)		
Balloon-expandable	12 (10.6%)	9 (15.3%)	1 (6.2%)	0		
Pre-dilation	101 (89.4%)	53 (89.8%)	15 (93.8%)	21 (95.5%)	0.617	0.940
Post-dilation	57 (50.4%)	25 (42.4%)	7 (43.8%)	11 (50%)	1.158	0.763
Permanent pacemaker implantation	15 (13.3%)	3 (5.1%)	1 (6.2%)	4 (18.2%)	4.202	0.206
Paravalvular leakage	50 (47.2%)	23 (39%)	7 (43.8%)	9 (40.9%)	0.472	0.925

RV-PA, right ventricular-pulmonary artery.

**Figure 2:**
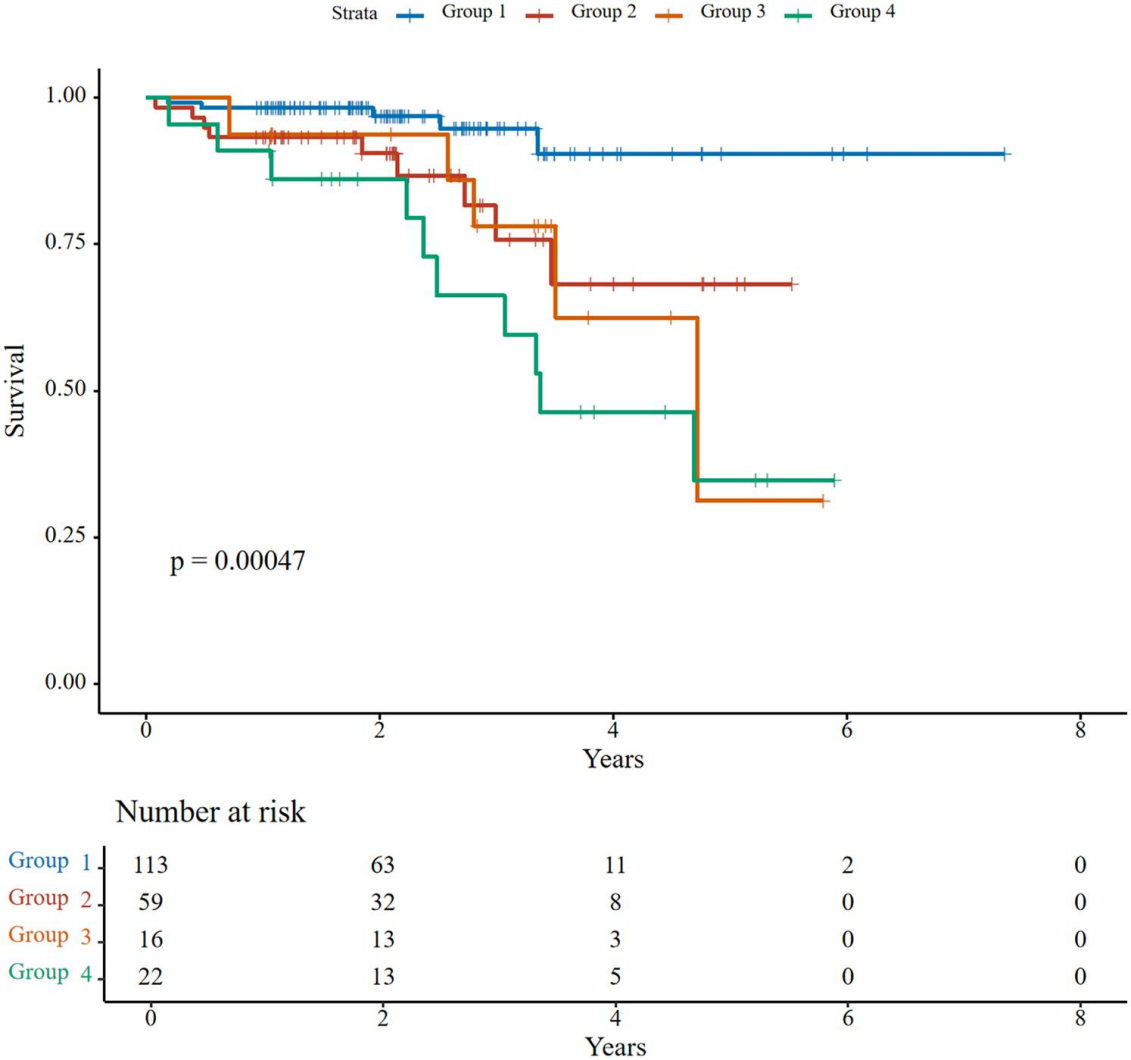
Kaplan–Meier survival curves for all-cause mortality stratified by right ventricular-pulmonary artery (RV-PA) coupling status.

Over a median follow-up of 26.1 months (IQR: 18.1–40.5), 29 patients (13.8%) experienced all-cause mortality. Kaplan–Meier analysis showed significant survival differences across groups (log-rank *P* < 0.001). The 5-year survival rate was highest in Group 1 (90%), followed by Group 2 (68%), while Groups 3 and 4 exhibited significantly lower survival rates (31% and 35%, respectively) (Figure 2). Group 4 had the highest mortality rate (45.5% vs. 4.4% in Group 1; HR = 5.61, 95% CI: 1.62–19.41, *P* = 0.006). No significant differences emerged in heart failure rehospitalization or NYHA functional class III/IV rates (all *P* > 0.05) (Table 4).

**Table 4:** Clinical outcomes during follow-up stratified by RV-PA coupling status.

Outcomes	Group 1 unchanged normal RV-PA coupling (*n* = 113)	Group 2 recovered RV-PA coupling (*n* = 59)	Group 3 new-onset RV-PA uncoupling (*n* = 16)	Group 4 persistent RV-PA uncoupling (*n* = 22)	*X*^2^/*H*	*P* value
All-cause mortality	5 (4.4%)	9 (15.3%)	5 (31.3%)	10 (45.5%)	27.563	**<0** **.** **001**
Heart failure rehospitalization	9 (8%)	7 (11.9%)	3 (18.8%)	6 (27.3%)	7.076	0.053
NYHA functional class III/IV	12 (10.6%)	8 (13.6%)	3 (18.8%)	5 (22.7%)	3.204	0.352

RV-PA, right ventricular-pulmonary artery.

Statistical significance at the 0.05 level is shown in bold type.

**Figure 3:**
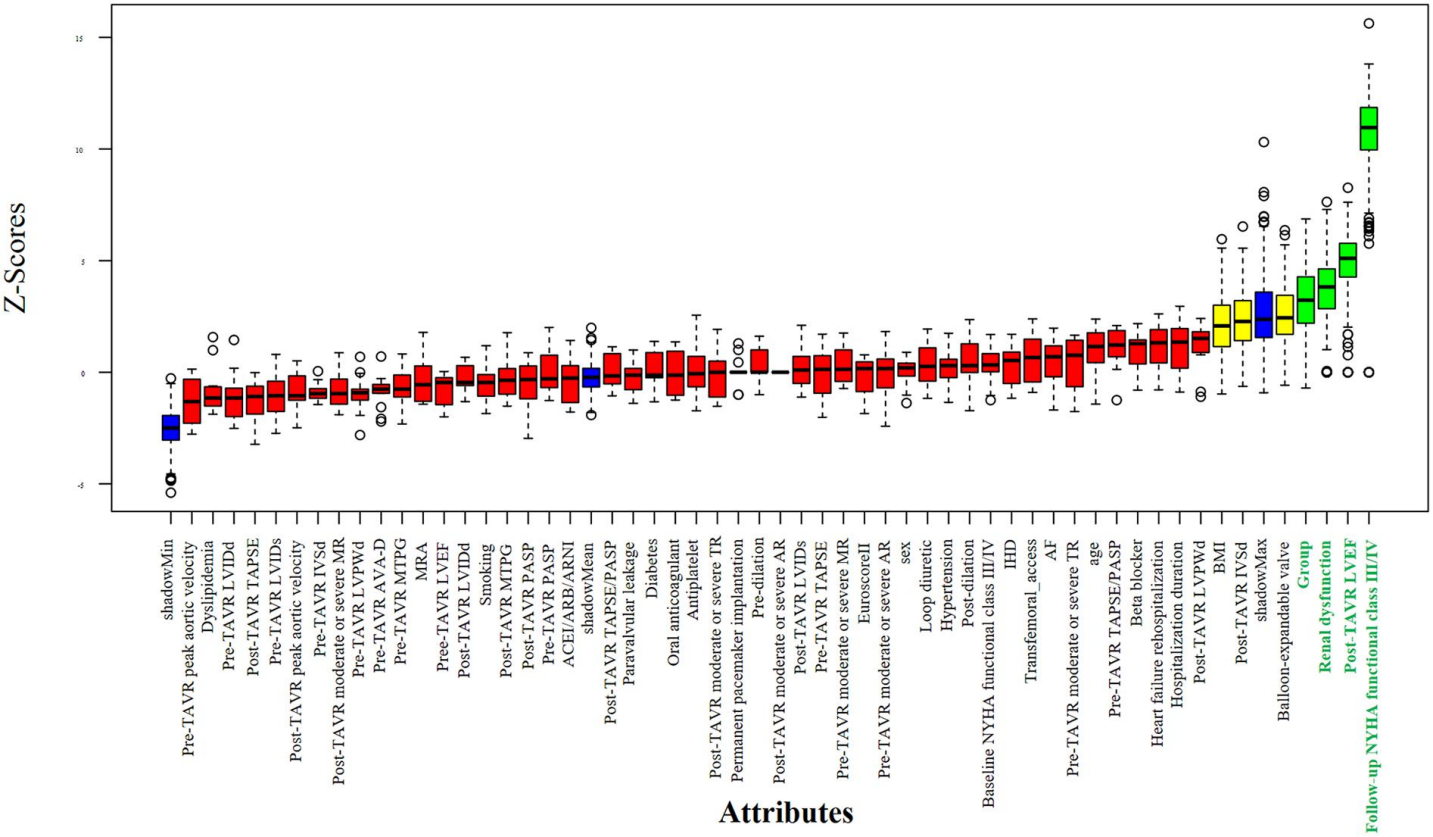
Feature selection using the Boruta algorithm for mortality predictors.

**Figure 4:**
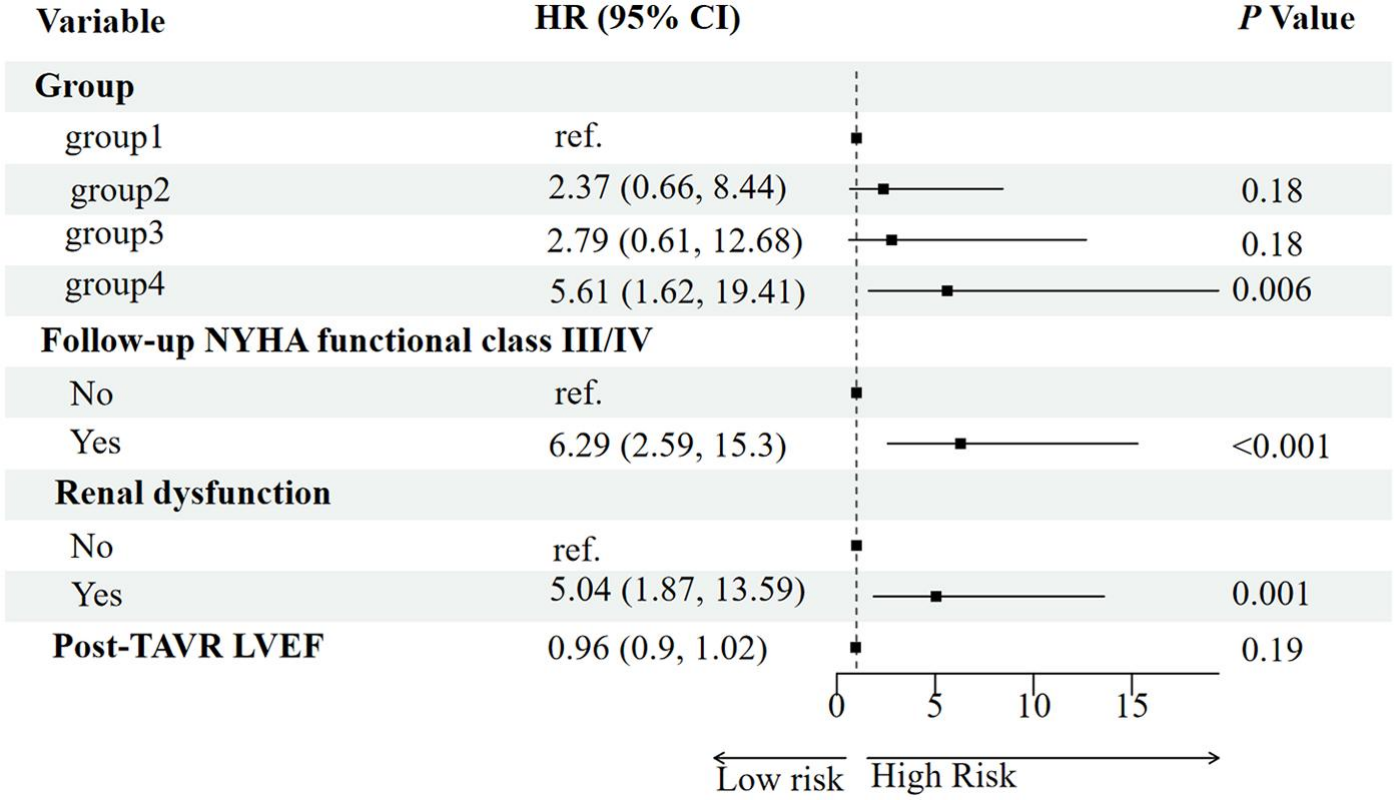
Forest plot of univariate analysis for predictors of all-cause mortality.

**Figure 5:**
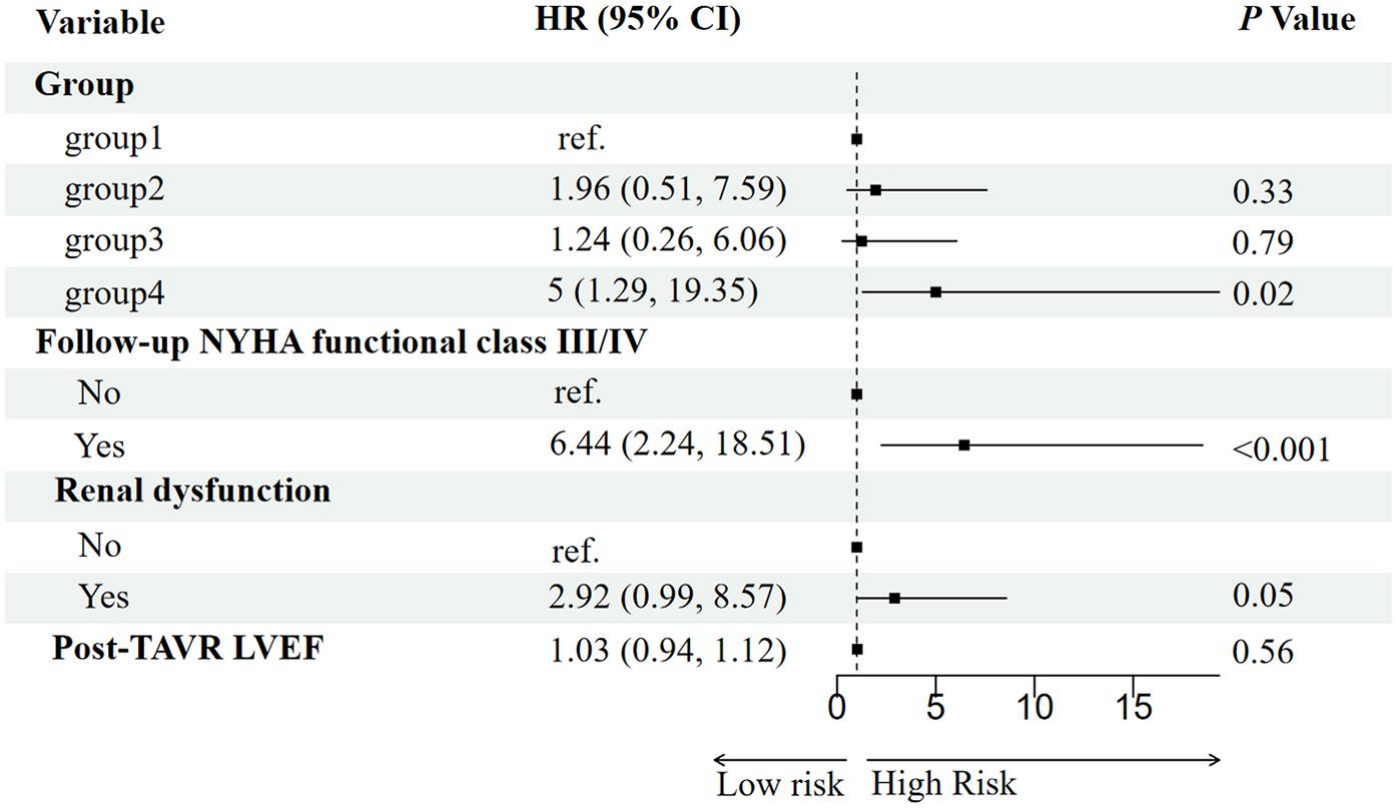
Forest plot of multivariable analysis for predictors of all-cause mortality.

Potential predictors of all-cause mortality were first screened using the Boruta algorithm, which identified four significant variables: RV-PA coupling group, post-TAVR LVEF, follow-up NYHA functional class, and baseline renal dysfunction (Figure 3). In univariable Cox regression analysis, persistent RV-PA uncoupling (Group 4), higher follow-up NYHA functional class, and baseline renal dysfunction were all significantly associated with increased mortality risk (Figure 4). To assess whether persistent RV-PA uncoupling remained an independent predictor after adjustment for other significant clinical factors, we performed a multivariable Cox regression analysis. After adjusting for age, renal dysfunction, and follow-up NYHA functional class, persistent RV-PA uncoupling (Group 4) remained strongly associated with a significantly increased risk of all-cause mortality (Adjusted HR: 5.00; 95% CI: 1.29–19.35; *P* = 0.02) (Figure 5).

## 4 Discussion

The present study describes the changes in RV-PA coupling between baseline and approximately 3-month echocardiography and their prognostic implications in patients with severe AS. The key findings highlight that both persistent and new-onset RV-PA uncoupling post-TAVR were associated with elevated mortality risk, consistent with prior evidence that RV-PA uncoupling serves as a predictor of adverse cardiovascular outcomes (11), and unresolved right ventricular dysfunction post-TAVR heralds reduced survival (7) Notably, our study adds granularity by demonstrating that even patients with new-onset uncoupling face substantial mortality risks (31.3%), approaching those with persistent dysfunction (45.5%). This suggests that dynamic RV-PA deterioration post-TAVR, regardless of baseline status, portends adverse outcomes. Patients with persistent uncoupling (Group 4) presented a distinct and more compromised clinical profile at baseline, characterized by a higher burden of atrial fibrillation, greater loop diuretic use, and a longer index hospitalization duration. This constellation of features is consistent with a generally sicker patient phenotype prior to the procedure, further supporting the notion that Group 4 represents a distinct high-risk subset requiring more intensive management.

Significant changes in RV-PA coupling were observed in this cohort. Among patients with baseline RV-PA uncoupling, the majority (72.8%) recovered normal coupling post-TAVR, whereas a smaller proportion (12.4%) of those with normal baseline coupling developed new-onset uncoupling. Mechanistically, the observed RV-PA coupling improvements in Group 2 likely reflect hemodynamic benefits of TAVR, including reduced left ventricular afterload and subsequent pulmonary pressure alleviation (13, 14). New-onset uncoupling may reflect transient hemodynamic stress post-TAVR. Persistent uncoupling was characterized by fixed, elevated afterload—evidenced by persistently high postoperative PASP—alongside a high burden of moderate-to-severe tricuspid regurgitation, despite showing LVEF improvement. This phenotype suggests underlying irreversible pulmonary vascular remodeling or intrinsic right ventricular dysfunction that fails to adapt even after TAVR (15). The consistent findings of both elevated PASP and significant TR, though methodologically linked, together indicate a phenotype of combined pulmonary hypertension and right-sided volume overload. Additionally, persistent or new-onset RV-PA uncoupling post-TAVR may be associated with complications such as cardiac amyloidosis (16) or left bundle branch block (LBBB) (17), which could exacerbate myocardial stiffness or ventricular dyssynchrony, further impairing right ventricular adaptation, further impairing right ventricular adaptation. The mortality rates of 15.3% in recovered coupling vs. 31.3% in new-onset uncoupling highlight the prognostic significance of serial RV-PA coupling assessments, emphasizing the necessity for continuous echocardiographic monitoring before and after TAVR.

While the improvement in RV-PA coupling was principally driven by a reduction in PASP post-TAVR, the TAPSE/PASP ratio provides critical prognostic information beyond afterload assessment alone. Whereas PASP solely reflects the magnitude of afterload burden, the TAPSE/PASP ratio integrates ventricular functional response, thereby distinguishing patients with compensated physiology from those with maladaptive right ventricular dysfunction. This composite measure of coupling efficiency offers superior risk stratification by identifying the high-risk phenotype of a ventricle unable to adapt to its hemodynamic load.

The sequential analysis—from feature selection to multivariable adjustment—consistently underscored the paramount importance of RV-PA coupling. The Boruta algorithm initially identified the RV-PA coupling group, post-TAVR LVEF, follow-up NYHA functional class, and baseline renal dysfunction as key variables associated with mortality. These factors align with established prognostic markers in TAVR populations. For instance, advanced NYHA functional class (18) is a classic predictor of mortality, reflecting compromised cardiac reserve and hemodynamic decompensation. Baseline renal dysfunction may exacerbate systemic inflammation and fluid retention, impairing cardiorenal interactions and increasing complication risks (19, 20).

The critical finding, however, emerged from the multivariable Cox regression. After adjusting for these clinically relevant confounders, persistent RV-PA uncoupling remained a strong and independent predictor of mortality. This indicates that its prognostic value is not merely a reflection of poor functional status (NYHA class) or comorbid renal impairment. The fact that the coupling group retained its significance in a model that included these powerful covariates significantly strengthens the argument for its independent role in risk stratification. This analysis solidifies the RV-PA coupling trajectory as a central determinant of post-TAVR survival, providing incremental prognostic information beyond traditional clinical parameters.

This study has several limitations. First, it is a retrospective observational study conducted at a single center with a relatively small sample size (*n* = 210). Over the nearly 6-year inclusion period, the average rate of patient inclusion was only about 3 patients per month. This low rate of case identification, while possibly reflecting stringent inclusion criteria, may introduce selection bias and limits the generalizability of our findings. Second, the exclusion of patients with incomplete echocardiographic data may have skewed the cohort toward healthier individuals. Third, changes in cardiac loading conditions driven by medications such as diuretics, renin-angiotensin-aldosterone system inhibitors, or pulmonary vasodilators could have influenced both PASP and right ventricular function, thereby potentially confounding the observed changes in TAPSE/PASP. Fourth, the classification of RV-PA coupling changes relied on only two timepoints (baseline and a single post-procedural echocardiogram). This approach cannot distinguish transient fluctuations from sustained changes. Therefore, our findings represent an initial assessment of early changes rather than a definitive characterization of long-term trajectories. Finally, the absence of invasive hemodynamic or advanced imaging data (e.g., cardiac MRI) restricts mechanistic insights into RV-PA coupling dynamics. Prospective, multicenter studies with systematic biomarker and advanced imaging data are needed to validate the prognostic role of RV-PA coupling trajectories and to guide targeted interventions in high-risk patients.

Our findings highlight that changes in RV-PA coupling were principally driven by alterations in PASP. This aligns with the pathophysiology of severe AS. While PASP quantifies the afterload burden, it cannot assess the right ventricular functional response.

## 5 Conclusion

This investigation establishes pre- to post-TAVR RV-PA coupling trajectories as pivotal prognostic determinants in severe AS. Specifically, persistent RV-PA uncoupling after TAVR is a strong predictor of increased mortality, confirming its critical role in risk stratification. The serial assessment of this parameter could help identify high-risk patients who may benefit from closer follow-up. Larger, prospective studies integrating biomarkers and advanced imaging are essential to validate these findings and elucidate the underlying mechanisms.

## Data Availability

The raw data supporting the conclusions of this article will be made available by the authors, without undue reservation.
